# The potential of respiration inhibition as a new approach to combat human fungal pathogens

**DOI:** 10.1007/s00294-019-01001-w

**Published:** 2019-06-06

**Authors:** Lucian Duvenage, Carol A. Munro, Campbell W. Gourlay

**Affiliations:** 1 Kent Fungal Group, School of Biosciences, University of Kent, Kent, CT2 9HY UK; 2 MRC Centre for Medical Mycology, Institute of Medical Sciences, University of Aberdeen, Foresterhill, Aberdeen, AB25 2ZD UK

**Keywords:** Mitochondria, Candida, Respiration, Respirometry, Metabolism, Yeast

## Abstract

The respiratory chain has been proposed as an attractive target for the development of new therapies to tackle human fungal pathogens. This arises from the presence of fungal-specific electron transport chain components and links between respiration and the control of virulence traits in several pathogenic species. However, as the physiological roles of mitochondria remain largely undetermined with respect to pathogenesis, its value as a potential new drug target remains to be determined. The use of respiration inhibitors as fungicides is well developed but has been hampered by the emergence of rapid resistance to current inhibitors. In addition, recent data suggest that adaptation of the human fungal pathogen, *Candida albicans*, to respiration inhibitors can enhance virulence traits such as yeast-to-hypha transition and cell wall organisation. We conclude that although respiration holds promise as a target for the development of new therapies to treat human fungal infections, we require a more detailed understanding of the role that mitochondria play in stress adaption and virulence.

## Introduction

Invasive fungal infections are a leading cause of morbidity and mortality in immunocompromised individuals. Among these, candidaemia is the most prevalent fungal bloodstream infection, with is accompanied by a high mortality rate of up to 40% (Pfaller and Diekema 2007). Patients in intensive care units are most susceptible to invasive *Candida* infections, and abdominal surgery and neutropenia are major risk factors. Colonisation of indwelling catheters by *Candida* or translocation from the gut to the bloodstream is also a frequent source of infection (Blumberg et al. 2001). Resistance can also be acquired through selection pressure in individual patients, particularly in cases of long-term use and when used as prophylaxis (Marr et al. 1998). Taken together, the evolution of resistance to current antifungals and the increasing involvement of inherently resistant species such as *Candida glabrata* (Sadeghi et al. 2018) and *Candida auris* (Sears and Schwartz 2017) in invasive candidiasis serves as an example that there is a need for the development of new antifungal targets.

Some of the novel antifungal agents currently under investigation target signal transduction pathways, iron metabolism and metabolic pathways such as the glyoxylate cycle (McCarthy et al. 2017). Several plant fungicides act by inhibiting components of the respiratory chain, but targeting of mitochondria has not yet been investigated as a therapy against invasive human fungal infections. Although the importance of mitochondrial function in fungal pathogenesis has been documented (Calderone et al. 2015), the conservation of the respiratory machinery in eukaryotes raises toxicity concerns for drug development and may in part explain why the influence of respiration in human fungal pathogens has remained an under-researched area. However, recent work has revealed the divergence of fungal respiratory chain components from those of the human host (She et al. 2015). A greater understanding of mitochondrial biology in invasive fungal pathogens such as *C. albicans* may expose weaknesses that can be exploited for anti-fungal development. Indeed, respiration inhibition has been shown to be effective in the management of malaria and *Pneumocystis* pneumonia.

## Inhibition of respiration in human fungal pathogens

**Fig. 1:**
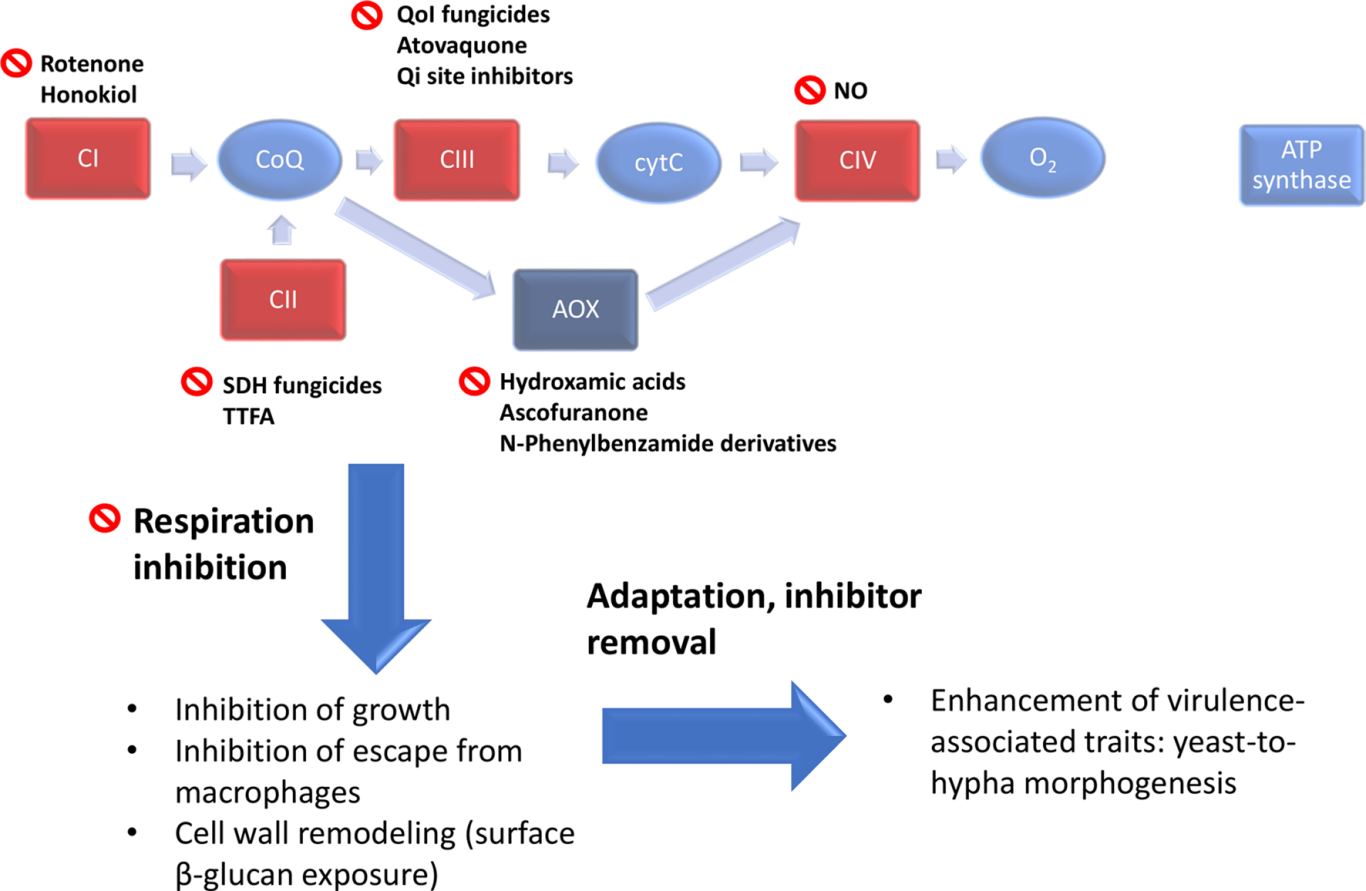
Inhibitors of the fungal electron transport chain. The majority of fungal pathogens possess the classical respiratory chain consisting of Complexes I–IV, as well as an alternative oxidase (AOX). Inhibitors of each component are shown. Respiration inhibition inhibits growth and leads to cell wall remodelling in *C. albicans* but enhances virulence if inhibition is relieved following adaptation. *SDH* succinate dehydrogenase, *TTFA* 2-thenoyltrifluoroacetone, *NO* nitric oxide

Most fungal pathogens possess a classical electron transport chain (ETC) consisting of Complexes I–IV, in addition to a cyanide-insensitive alternative oxidase (AOX) (Fig. 1). The notable exception to this being *Candida glabrata* which, like *Saccharomyces cerevisiae*, does not contain a multi-subunit Complex I or an AOX enzyme activity. Evidence for a third *“*parallel” ETC pathway has been described in *Candida parapsilosis* and *C. albicans* which represents approximately 10% of total respiration capacity (Milani et al. 2001; Duvenage et al. 2019). Several pathogenic fungi depend on oxidative phosphorylation for virulence. For example, respiration deficiency leads to attenuated virulence in the fungal pathogens *C. albicans* (Aoki et al. 1990), *C. glabrata* (Brun et al. 2005) and *Aspergillus fumigatus* (Grahl et al. 2012)*.* The links between respiration and virulence are not well understood but may include the energy requirement for adaptation to the host environment, the involvement of respiration in cellular remodelling processes such as morphogenesis or the role of mitochondria in stress signalling. For example, high ATP levels resulting from respiratory activity have been shown to be crucial for *C. albicans* yeast cells to switch to hyphal growth via Ras1/cAMP/PKA signalling (Grahl et al. 2015). In addition, increased ATP from respiration has been shown to be important for morphogenesis during the catabolism of morphogenic amino acids, and is an important feature of escape of *C. albicans* from macrophages (Silao et al. 2019).

The use of respiratory chain inhibitors can replicate the in vitro growth defects of respiration-deficient mutants. For example, in *C. albicans,* inhibitors such as Antimycin A and cyanide lead to inhibition of growth, and increased oxidative stress (Ruy et al. 2006). Similarly, phenolics that inhibit mitochondrial function inhibit the growth of *A. fumigatus* (Brun et al. 2005)*.* These observations suggest that a pharmacological approach to inhibition of respiration may prove effective as an approach to treating fungal infection.

### Complex I inhibition

Complex I (NADH:ubiquinone oxidoreductase) is present in most fungal pathogens (although it is absent in some yeasts such as *S. cerevisiae* and *C. glabrata*). Recent work has identified important Complex I regulatory proteins in *C. albicans* as well as subunits of the complex itself to be fungal specific (She et al. 2015). Deletion of these proteins leads to deficiencies in respiration and virulence, making them attractive drug targets. (She et al. 2015, 2018). The Complex I subunits Nuo1 and Nuo2 are conserved in several fungal pathogens including other *Candida* species, *A. fumigatus* and *Cryptococcus neoformans* (Li and Calderone 2017)*.* Dysfunction of Complex I is one of the main sources of mitochondrial ROS accumulation, which can promote fungal cell death (Li et al. 2011). Therefore, inhibitors of fungal Complex I have the potential to have both a fungistatic effect, by limiting ATP production, as well as a fungicidal activity via increased ROS levels. This potential was demonstrated by the fungicidal effects of honokiol, derived from *Magnolia officinalis*, caused through inhibition of Complex I (Sun et al. 2017). The putative mitochondrial fission inhibitor mdivi-1, which has been shown to inhibit mammalian Complex I as a secondary target, prevents hyphal growth in *C. albicans* (Koch and Traven 2019)*.* However, it is suggested that this mechanism of action may not be Complex I dependent, as the effects of mdivi-1 were replicated in the respiration-deficient *nuo1*Δ mutant (Koch et al. 2018). Instead, the authors suggest that mdivi-1 may regulate Nrg1, an important repressor of filamentous growth (Su et al. 2018).

### Complex II inhibition

Complex II, succinate:ubiquinone oxidoreductase, transfers succinate-derived electrons directly to the ubiquinone pool of the respiratory chain and not to soluble NAD+ intermediates. Its activity connects respiration to the TCA cycle, which supplies intermediates for biosynthesis of macromolecules in addition to its role in supporting respiration. Inhibitors of Complex II include 2-thenoyltrifluoroacetone (TTFA) which inhibits its quinone reduction activity, and 3-nitropropionate, a succinate analogue which inhibits succinate oxidation activity. Succinate dehydrogenase inhibitors are a fast-growing class of fungicides against plant fungal pathogens, which act by binding the (Qp) ubiquinone binding site (Sierotzki and Scalliet 2013; Guo et al. 2019; Amiri et al. 2019). The role of Complex II in the virulence of human fungal pathogens is not well understood, and thus its inhibition has not yet been explored as an antifungal therapy. However, there is evidence that Complex II function is important for morphogenesis, as inhibition of quinone reduction activity with TTFA was shown to completely inhibit filamentation in *C. albicans* (Watanabe et al. 2006).

### Complex III inhibition

Complex III, the cytochrome *bc1* complex, transfers electrons from the ubiquinol pool to cytochrome *c*. Along with Complex I, Complex III is a major source of mitochondrial ROS accumulation (Meunier et al. 2013). Inhibition of Complex III with antimycin A in *C. albicans* was effective in decreasing proliferation, which in part may be the result of an increase in oxidative stress (Ruy et al. 2006). Inhibitors of Complex III may bind to the ubiquinol oxidation (Qo) or ubiquinone reduction (Qi) site. QoI fungicides inhibit mitochondrial respiration in plant pathogenic fungi by binding to the Qo site of Complex III (Fernández-Ortuño et al. 2008). Although effective, resistance to QoI fungicides is a growing problem, mediated by both acquisition of mutations in the cytochrome *b* gene as well as the increased activity of AOX enzymes (Fernández-Ortuño et al. 2008). The hydroxynaphthoquinone atovaquone also acts by inhibiting the Qo site and has been shown to be effective against malaria parasites as well as the opportunistic fungal pathogen *Pneumocystis jirovecii* (Fisher and Meunier 2008). One study reports the resistance of *C. albicans* to Atovaquone and that it is not effective in repressing glucose-dependent growth. However, the report also describes its efficacy in inhibiting respiration to a degree comparable to antimycin A (Minagawa et al. 2010).The failure of Atovaquone to suppress *C. albicans* growth in this case may be due to induction of alternative respiration. The efficacy of Atovaquone against *Plasmodium falciparum* was increased when used in combination with AOX inhibitors (Murphy and Lang-unnasch 1999), although whether this holds true for fungal pathogens is unknown*.* A recent study reported a fungal-specific inhibitor of Complex III in *C. albicans* resulting from a screen of a 300,000-compound library. The authors identified a compound that could specifically inhibit fungal Complex III by binding to the Qo site, that was synergistic with fluconazole (Vincent et al. 2016). Inhibition of Complex III through use of ubiquinone analogues is also an attractive strategy, as suitable compounds have the potential to inhibit the activities of both Complex III and AOX, leading to complete inhibition of respiration.

### Complex IV inhibition

Complex IV (cytochrome *c* oxidase) is the terminal oxidase of the classical ETC, reducing oxygen to water. It belongs to the heme–copper oxidase superfamily and in *S. cerevisiae,* it consists of 11 subunits (Geier et al. 1995). The conservation of Complex IV between mammals and fungi has made it less attractive as an antifungal target. However, it has long been known that Complex IV of microbes is susceptible to inhibition by nitric oxide (NO) and in recent years, the applications of NO against pathogenic fungi have been an active area of research (Macherla et al. 2012; Ahmadi et al. 2016; Mordorski et al. 2017). NO binds the oxygen-binding site, and can either be reversible and competitive with oxygen, or irreversible, with higher NO– and lower oxygen concentrations favouring the latter (Brown and Borutaite 2002). Due to its vasodilation effect, NO may not be suitable for systemic fungal infections, unless specific targeting and controlled release systems can be developed. In addition, achieving a sustained high level of NO—given its very short half-life in vivo—and effective targeting of NO donors to organs affected by deep-seated fungal infections poses a considerable challenge. Instead, NO may be best employed as a preventative measure. For example, as indwelling catheters are a major cause of disseminated *Candida* infections, medical devices which release NO could be effective in preventing such infections, as has been investigated for nitric oxide-charged catheters (Margel et al. 2017).

## Targeting alternative oxidase function

In addition to the classical ETC, many pathogenic fungi possess a cyanide-insensitive alternative pathway, not found in mammals, which permits respiration when the classical chain is inhibited (Huh and Kang 1999). AOX activity is not coupled to the generation of a proton gradient across the mitochondrial membrane, and thus alternate respiration produces significantly less ATP than classical oxidative phosphorylation (Helmerhorst et al. 2002). This suggests that AOX-based respiration does not have a key role in energy production but permits respiration under conditions of classical chain inhibition. Although alternative respiration is energetically less favourable, it allows respiration to continue upon inhibition of the classical electron transport system, thus maintaining essential metabolic functions of the mitochondrial compartment and supporting viability. Therefore, a combination of classical- and alternative respiratory pathway inhibitors may be the most effective antifungal strategy and limit the development of resistance*.*

AOX is inhibited by hydroxamic acids such as salicylhydroxamic acid (SHAM), n-propyl gallate and the natural antibiotic ascofuranone, a potent inhibitor of trypanosome AOX (Menzies et al. 2018). The importance of AOX in morphogenesis and resistance to oxidative stress has been demonstrated in several fungal pathogens, including *A. fumigatus* (Magnani et al. 2008)*, C. neoformans* (Akhter et al. 2003) and *Paracoccidioides brasiliensis* (Martins et al. 2011)*.* However, despite these important functions, reports suggest that AOX is dispensable for virulence in some fungal pathogens, including *C. albicans* (Huh and Kang 2001) and *A. fumigatus* (Grahl et al. 2012)*.* Therefore, AOX inhibitors may not be universally successful as antifungals, at least not as a monotherapy. It is likely that inhibition of AOX could be effective in combination with classical ETC inhibitors or antifungals which induce oxidative stress, although this has not yet been tested in vivo due to a lack of suitable fungal and highly specific AOX inhibitors.

AOX inhibitors for trypanosomiasis have not yet reached the clinical trial stage but research in this area seems promising, with the discovery of new inhibitors such as ascofuranone as well as optimisation of existing inhibitors (Ott et al. 2006). Development of fungal AOX inhibitors is hampered by the fact that so far, *Trypanosoma brucei* AOX is the only alternative oxidase protein structure available. Nevertheless, optimisation of *N*-phenylbenzamide derivatives showed promise against the phytopathogen *Moniliophthora perniciosa* (Barsottini et al. 2018).

In our recent study of the response to *C. albicans* to respiration inhibition with the NO donor, sodium nitroprusside (SNP), and the alternative oxidase inhibitor, salicylhydroxamic acid (SHAM), we found that cells which had been exposed to inhibitors showed a more rapid transition to hyphal growth when inhibition was relieved (Duvenage et al. 2019). SNP + SHAM-pretreated *C. albicans* exhibited increased virulence in the mouse model of systemic candidiasis, with the kidneys of infected animals showing a higher fungal burden and an increased immune infiltrate compared to infection with untreated *C. albicans*. Part of the adaptation to respiration inhibitors includes the induction of Aox2 levels which leads to an increase in alternative respiration capacity. We found that AOX has an important role in this hyphal switching phenotype, as we did not observe increased hyphal switching, as seen in the wild type, in an *aox*Δ mutant following SNP + SHAM pretreatment. This highlights a requirement for a better understanding of the role of mitochondria in stress signalling, if respiration inhibitors are to be developed as successful therapeutics.

## Respiration inhibition can be synergistic with current antifungals

Due to the connection between mitochondrial function and other cell processes such and ergosterol synthesis and cell wall maintenance (Dagley et al. 2011), respiration inhibitors have the potential to enhance the effects of current antifungals. Fungal-specific inhibitors of Complex III were identified in a screen to identify compounds which reverse azole resistance (Vincent et al. 2016). In addition to reversing fluconazole resistance, one of the compounds (Inz-5) also enhanced the ability of macrophages to control growth of *C. albicans*, demonstrating that respiration inhibitors alone may be effective by complementing the immune response. Inhibition of mitochondrial aerobic respiratory metabolism with tetrandrine caused increased sensitivity to fluconazole (Guo et al. 2014). The authors suggest that decreased ATP levels may inhibit resistance by decreasing the action of drug efflux pumps. Lastly, there is some evidence that the alternative oxidase can contribute to fluconazole resistance. A combination of SHAM and fluconazole showed a degree of synergism against *C. albicans* (Yan et al. 2009).

Respiration-deficient mutants show a defect in ergosterol biosynthesis, and thus are sensitive to polyenes such as Amphotericin B (Geraghty and Kavanagh 2003a). Respiration inhibition can replicate this effect: erythromycin, an inhibitor of cytochrome synthesis (particularly cytochrome aa_3_ of Complex IV), enhances sensitivity to Amphotericin B (Geraghty and Kavanagh 2003b). Although the links between respiration and lipid metabolism are well known, the effect of respiration inhibition on the fungal cell wall is an under-studied area. Inhibition of Complex III and the alternative oxidase in combination enhances susceptibility to caspofungin in *C. parapsilosis* (Chamilos et al. 2006). However, in our recent study of *C. albicans,* respiration inhibition with SNP and SHAM was found to decrease caspofungin susceptibility. This suggests that inhibition of different classical respiratory chain components may have different outcomes on cell wall targeting drug susceptibilities, or that the connection between respiration and cell wall regulation may vary by species.

In summary, due to the connection of mitochondria to pathogenesis, cell wall regulation and lipid metabolism, respiration inhibitors may prove to be effective against fungal pathogens either in isolation or in combination with current antifungals. However, the conservation of the respiratory machinery in eukaryotes and the robust and adaptive nature of fungal respiration is a challenge for drug development. The discovery and characterisation of fungal-specific respiratory chain components are needed, together with a deeper understanding of the roles of those already characterised, such as the AOX.

## References

[CR1] Ahmadi M, Lee HH, Sanchez DA (2016). Sustained nitric oxide releasing nanoparticles induce cell death in *Candida albicans* yeast and hyphal cells preventing biofilm formation in vitro and in a rodent central venous catheter model. Antimicrob Agents Chemother.

[CR2] Akhter S, McDade HC, Gorlach JM (2003). Role of alternative oxidase gene in pathogenesis of *Cryptococcus neoformans*. Infect Immun.

[CR3] Amiri A, Zuniga AI, Cordova LG, Peres NA (2019). The importance of selecting appropriate rotation and tank-mix partners for novel SDHIs to enhance botrytis fruit rot control in strawberry. Plant Dis.

[CR4] Aoki S, Ito-Kuwa S, Nakamura Y, Masuhara T (1990). Comparative pathogenicity of a wild-type strain and respiratory mutants of *Candida albicans* in mice. Zentralbl Bakteriol.

[CR5] Barsottini MRO, Pires BA, Vieira ML (2018). Synthesis and testing of novel alternative oxidase (AOX) inhibitors with antifungal activity against *Moniliophthora perniciosa* (Stahel), the causal agent of witches’ broom disease of cocoa, and other phytopathogens. Pest Manag Sci.

[CR6] Blumberg HM, Jarvis WR, Soucie JM (2001). Risk factors for candidal bloodstream infections in surgical intensive care unit patients: the NEMIS prospective multicenter study. Clin Infect Dis.

[CR7] Brown GC, Borutaite V (2002). Nitric oxide inhibition of mitochondrial respiration and its role in cell death. Free Radic Biol Med.

[CR8] Brun S, Dalle F, Saulnier P (2005). Biological consequences of petite mutations in *Candida glabrata*. J Antimicrob Chemother.

[CR9] Calderone R, Li D, Traven A (2015). System-level impact of mitochondria on fungal virulence: to metabolism and beyond. FEMS Yeast Res.

[CR10] Chamilos G, Lewis RE, Kontoyiannis DP (2006). Inhibition of *Candida parapsilosis* mitochondrial respiratory pathways enhances susceptibility to caspofungin. Antimicrob Agents Chemother.

[CR11] Dagley MJ, Gentle IE, Beilharz TH (2011). Cell wall integrity is linked to mitochondria and phospholipid homeostasis in *Candida albicans* through the activity of the post-transcriptional regulator Ccr4-Pop2. Mol Microbiol.

[CR12] Duvenage L, Walker LA, Bojarczuk A (2019). Inhibition of classical and alternative modes of respiration in *Candida albicans* leads to cell wall remodeling and increased macrophage recognition. MBio.

[CR13] Fernández-Ortuño D, Torés JA, De Vicente A, Pérez-García A (2008). Mechanisms of resistance to QoI fungicides in phytopathogenic fungi. Int Microbiol.

[CR14] Fisher N, Meunier B (2008). Molecular basis of resistance to cytochrome bc1 inhibitors. FEMS Yeast Res.

[CR15] Geier BM, Schägger H, Ortwein C (1995). Kinetic properties and ligand binding of the eleven-subunit cytochrome-c oxidase from *Saccharomyces cerevisiae* isolated with a novel large-scale purification method. Eur J Biochem.

[CR16] Geraghty P, Kavanagh K (2003). Disruption of mitochondrial function in *Candida albicans* leads to reduced cellular ergosterol levels and elevated growth in the presence of amphotericin B. Arch Microbiol.

[CR17] Geraghty P, Kavanagh K (2003). Erythromycin, an inhibitor of mitoribosomal protein biosynthesis, alters the amphotericin B susceptibility of *Candida albicans*. J Pharm Pharmacol.

[CR18] Grahl N, Dinamarco TM, Willger SD (2012). *Aspergillus fumigatus* mitochondrial electron transport chain mediates oxidative stress homeostasis, hypoxia responses and fungal pathogenesis. Mol Microbiol.

[CR19] Grahl N, Demers EG, Lindsay AK (2015). Mitochondrial activity and Cyr1 are key regulators of Ras1 activation of *C. albicans* virulence pathways. PLOS Pathog.

[CR20] Guo H, Xie SM, Li SX (2014). Synergistic mechanism for tetrandrine on fluconazole against *Candida albicans* through the mitochondrial aerobic respiratory metabolism pathway. J Med Microbiol.

[CR21] Guo X, Zhao B, Fan Z (2019). Discovery of novel thiazole carboxamides as antifungal succinate dehydrogenase inhibitors. J Agric Food Chem.

[CR22] Helmerhorst EJ, Murphy MP, Troxler RF, Oppenheim FG (2002). Characterization of the mitochondrial respiratory pathways in *Candida albicans*. Biochim Biophys Acta.

[CR23] Huh WK, Kang SO (1999). Molecular cloning and functional expression of alternative oxidase from *Candida albicans*. J Bacteriol.

[CR24] Huh WK, Kang SO (2001). Characterization of the gene family encoding alternative oxidase from *Candida albicans*. Biochem J.

[CR25] Koch B, Traven A (2019). Mdivi-1 and mitochondrial fission: recent insights from fungal pathogens. Curr Genet.

[CR26] Koch B, Barugahare AA, Lo TL (2018). A metabolic checkpoint for the yeast-to-hyphae developmental switch regulated by endogenous nitric oxide signaling. Cell Rep.

[CR27] Li D, Calderone R (2017). Exploiting mitochondria as targets for the development of new antifungals. Virulence.

[CR28] Li D, Chen H, Florentino A (2011). Enzymatic dysfunction of mitochondrial Complex I of the *Candida albicans* goa1 mutant is associated with increased reactive oxidants and cell death. Eukaryot Cell.

[CR29] Macherla C, Sanchez DA, Ahmadi MS (2012). Nitric oxide releasing nanoparticles for treatment of *Candida albicans* burn infections. Front Microbiol.

[CR30] Magnani T, Soriani FM, Martins VDP (2008). Silencing of mitochondrial alternative oxidase gene of *Aspergillus fumigatus* enhances reactive oxygen species production and killing of the fungus by macrophages. J Bioenerg Biomembr.

[CR31] Margel D, Mizrahi M, Regev-Shoshani G (2017). Nitric oxide charged catheters as a potential strategy for prevention of hospital acquired infections. PLoS ONE.

[CR32] Marr KA, Lyons CN, Rustad TR (1998). Rapid, transient fluconazole resistance in *Candida albicans* is associated with increased mRNA levels of CDR [published erratum appears in Antimicrob Agents Chemother 1999 Feb; 43(2):438]. Antimicrob Agents Chemother.

[CR33] Martins VP, Dinamarco TM, Soriani FM (2011). Involvement of an alternative oxidase in oxidative stress and mycelium-to-yeast differentiation in *Paracoccidioides brasiliensis*. Eukaryot Cell.

[CR34] McCarthy MW, Kontoyiannis DP, Cornely OA (2017). Novel agents and drug targets to meet the challenges of resistant fungi. J Infect Dis.

[CR35] Menzies SK, Tulloch LB, Florence GJ, Smith TK (2018). The trypanosome alternative oxidase: a potential drug target?. Parasitology.

[CR36] Meunier B, Fisher N, Ransac S (2013). Respiratory complex III dysfunction in humans and the use of yeast as a model organism to study mitochondrial myopathy and associated diseases. Biochim Biophys Acta Bioenerg.

[CR37] Milani G, Jarmuszkiewicz W, Sluse-Goffart CM (2001). Respiratory chain network in mitochondria of *Candida parapsilosis*: ADP/O appraisal of the multiple electron pathways. FEBS Lett.

[CR38] Minagawa N, Uehara M, Seki S (2010). Effects of combined addition of atovaquone and lithium on the in vitro cell growth of the pathogenic yeast *Candida albicans*. Yakugaku Zasshi.

[CR39] Mordorski B, Costa-Orlandi CB, Baltazar LM (2017). Topical nitric oxide releasing nanoparticles are effective in a murine model of dermal *Trichophyton rubrum* dermatophytosis. Nanomedicine Nanotechnology, Biol Med.

[CR40] Murphy AD, Lang-unnasch N (1999). Alternative oxidase inhibitors potentiate the activity of atovaquone against *Plasmodium falciparum*. Antimicrob Agents Chemother.

[CR41] Ott R, Chibale K, Anderson S (2006). Novel inhibitors of the trypanosome alternative oxidase inhibit *Trypanosoma brucei brucei* growth and respiration. Acta Trop.

[CR42] Pfaller MA, Diekema DJ (2007). Epidemiology of invasive candidiasis: a persistent public health problem. Clin Microbiol Rev.

[CR43] Ruy F, Vercesi AE, Kowaltowski AJ (2006). Inhibition of specific electron transport pathways leads to oxidative stress and decreased *Candida albicans* proliferation. J Bioenerg Biomembr.

[CR44] Sadeghi G, Ebrahimi-Rad M, Mousavi SF (2018). Emergence of non-*Candida albicans* species: epidemiology, phylogeny and fluconazole susceptibility profile. J Mycol Med.

[CR45] Sears D, Schwartz BS (2017). *Candida auris*: an emerging multidrug-resistant pathogen. Int J Infect Dis.

[CR46] She X, Khamooshi K, Gao Y (2015). Fungal-specific subunits of the *Candida albicans* mitochondrial complex I drive diverse cell functions including cell wall synthesis. Cell Microbiol.

[CR47] She X, Zhang P, Gao Y (2018). A mitochondrial proteomics view of complex I deficiency in *Candida albicans*. Mitochondrion.

[CR48] Sierotzki H, Scalliet G (2013). A review of current knowledge of resistance aspects for the next-generation succinate dehydrogenase inhibitor fungicides. Phytopathology.

[CR49] Silao FGS, Ward M, Ryman K (2019). Mitochondrial proline catabolism activates Ras1/cAMP/PKA-induced filamentation in *Candida albicans*. PLoS Genet.

[CR50] Su C, Yu J, Lu Y (2018). Hyphal development in *Candida albicans* from different cell states. Curr Genet.

[CR51] Sun L, Liao K, Wang D (2017). Honokiol induces superoxide production by targeting mitochondrial respiratory chain complex I in *Candida albicans*. PLoS ONE.

[CR52] Vincent BM, Scherz-Shouval R, Tidor B (2016). A fungal-selective cytochrome bc1 inhibitor impairs virulence and prevents the evolution of drug resistance. Cell Chem Biol.

[CR53] Watanabe T, Ogasawara A, Mikami T, Matsumoto T (2006). Hyphal formation of *Candida albicans* is controlled by electron transfer system. Biochem Biophys Res Commun.

[CR54] Yan L, Li M, Cao Y (2009). The alternative oxidase of *Candida albicans* causes reduced fluconazole susceptibility. J Antimicrob Chemother.

